# Prevention of tuberculosis in household members: estimates of children eligible for treatment

**DOI:** 10.2471/BLT.18.218651

**Published:** 2019-05-28

**Authors:** Yohhei Hamada, Philippe Glaziou, Charalambos Sismanidis, Haileyesus Getahun

**Affiliations:** a Global Tuberculosis Programme, World Health Organization, 20 avenue Appia, 1211 Geneva 27, Switzerland.

## Abstract

**Objective:**

To estimate of the number of children younger than 5 years who were household contacts of people with tuberculosis and were eligible for tuberculosis preventive treatment in 2017.

**Methods:**

To estimate the number of eligible children, we obtained national values for the number of notified cases of bacteriologically confirmed pulmonary tuberculosis in 2017, the proportion of the population younger than 5 years in 2017 and average household size from published sources. We obtained global values for the number of active tuberculosis cases per household with an index case and for the prevalence of latent tuberculosis infection among children younger than 5 years who were household contacts of a tuberculosis case through systematic reviews, meta-analysis and Poisson regression models.

**Findings:**

The estimated number of children younger than 5 years eligible for tuberculosis preventive treatment in 2017 globally was 1.27 million (95% uncertainty interval, UI: 1.24–1.31), which corresponded to an estimated global coverage of preventive treatment in children of 23% at best. By country, the estimated number ranged from less than one in the Bahamas, Iceland, Luxembourg and Malta to 350 000 (95% UI: 320 000–380 000) in India. Regionally, the highest estimates were for the World Health Organization (WHO) South-East Asia Region (510 000; 95% UI: 450 000–580 000) and the WHO African Region (470 000; 95% UI: 440 000–490 000).

**Conclusion:**

Tuberculosis preventive treatment in children was underutilized globally in 2017. Treatment should be scaled up to help eliminate the pool of tuberculosis infection and achieve the End TB Strategy targets.

## Résumé

**Objectif:**

Estimer le nombre d'enfants de moins de 5 ans qui étaient en contact avec des membres de la famille atteints de tuberculose et qui étaient éligibles à un traitement préventif de cette maladie en 2017.

**Méthodes:**

Pour estimer le nombre d'enfants éligibles, nous nous sommes procuré, à partir de diverses publications, les valeurs nationales correspondant au nombre de cas signalés de tuberculose pulmonaire confirmée par des analyses bactériologiques en 2017, à la part de la population âgée de moins de 5 ans en 2017 et à la taille moyenne des familles. Nous nous sommes procuré, au moyen d'une revue systématique, d'une méta-analyse et de modèles de régression de Poisson, les valeurs mondiales correspondant au nombre de cas de tuberculose active par foyer avec cas de référence et à la prévalence de l'infection tuberculeuse latente chez les enfants de moins de 5 ans qui étaient en contact avec un membre de la famille atteint de tuberculose.

**Résultats:**

Le nombre estimé d'enfants de moins de 5 ans éligibles à un traitement préventif de la tuberculose dans le monde en 2017 était de 1,27 million (intervalle d'incertitude de 95%, II: 1,24–1,31), soit une couverture mondiale de traitement préventif chez les enfants estimée à 23% au mieux. Par pays, le nombre estimé allait de moins d'un aux Bahamas, en Islande, au Luxembourg et à Malte à 350 000 (II 95%: 320 000–380 000) en Inde. Au niveau des régions, les estimations les plus élevées se retrouvaient dans la Région OMS de l'Asie du Sud-Est (510 000; II 95%: 450 000–580 000) et la Région africaine de l'OMS (470 000; II 95%: 440 000–490 000).

**Conclusion:**

Au niveau mondial, le traitement préventif de la tuberculose chez les enfants était sous-utilisé en 2017. Il faudrait intensifier le recours au traitement afin d'éliminer les foyers de tuberculose et d'atteindre les objectifs de la Stratégie de l'OMS pour mettre fin à la tuberculose.

## Resumen

**Objetivo:**

Estimar el número de niños menores de cinco años que tuvieron contacto con personas con tuberculosis en sus hogares y que eran elegibles para el tratamiento preventivo de la tuberculosis en 2017.

**Métodos:**

Para estimar el número de niños elegibles, se obtuvieron valores nacionales para el número de casos notificados de tuberculosis pulmonar bacteriológicamente confirmada en 2017, la proporción de la población menor de 5 años en 2017 y el tamaño promedio del hogar de fuentes publicadas. Se obtuvieron valores globales para el número de casos de tuberculosis activa por hogar con un caso índice y para la prevalencia de infección de tuberculosis latente entre los niños menores de 5 años que estaban en contacto con un caso de tuberculosis en el hogar mediante las revisiones sistemáticas, el metanálisis y los modelos de regresión de Poisson.

**Resultados:**

El número estimado de niños menores de 5 años elegibles para el tratamiento preventivo de la tuberculosis en 2017 a nivel mundial fue de 1,27 millones (intervalo de incertidumbre del 95 %, IU: 1,24-1,31), lo que corresponde a una cobertura mundial estimada de tratamiento preventivo en niños del 23 % en el mejor de los casos. Por país, el número estimado oscila entre menos de uno en las Bahamas, Islandia, Luxemburgo y Malta y 350 000 (95 % UI: 320 000-380 000) en la India. A nivel regional, las estimaciones más elevadas correspondieron a la Región de Asia Sudoriental de la Organización Mundial de la Salud (OMS) (510 000; IC del 95 %: 450 000-580 000) y a la Región Africana de la OMS (470 000; IC del 95 %: 440 000-490 000).

**Conclusión:**

El tratamiento preventivo de la tuberculosis en los niños fue utilizado muy poco a nivel mundial en 2017. El tratamiento debe ampliarse para ayudar a eliminar el conjunto de infecciones de tuberculosis y alcanzar los objetivos de la Estrategia de Fin a la Tuberculosis.

## ملخص

**الغرض:**

تقدير عدد الأطفال الذين تقل أعمارهم عن 5 سنوات والذين كانوا يقيمون في منزل واحد مع أشخاص مصابين بالسل، وكانوا مؤهلين للحصول على علاج وقائي من السل في عام 2017.

**الطريقة:**

لتقدير عدد الأطفال المؤهلين، حصلنا على قيم وطنية لعدد الحالات المبلغ عن إصابتها بمرض السل الرئوي المؤكد جرثوميًا في عام 2017، ونسبة السكان الذين تقل أعمارهم عن 5 سنوات في عام 2017، ومتوسط ​​حجم الأسرة من المصادر المنشورة. كما حصلنا على قيم عالمية لعدد حالات الإصابة بالسل النشطة لكل منزل مع حالة كمؤشر، وكذلك قيم لانتشار عدوى السل الكامنة بين الأطفال الذين تقل أعمارهم عن 5 سنوات والذين كانوا يقيمون في منزل واحد من حالة مرض بالسل من خلال المراجعات المنهجية، والتحليل التلوي، ونماذج التحوف لبواسون.

**النتائج:**

العدد التقديري للأطفال الذين تقل أعمارهم عن 5 سنوات، والمؤهلين للعلاج الوقائي من السل في عام 2017 على مستوى العالم، كان 1.27 مليون (فاصل عدم الثقة: 95%، 1.24 إلى 1.31)، وهو ما يتوافق مع تغطية عالمية تقديرية للعلاج الوقائي في الأطفال بنسبة 23% في أحسن الأحوال.حسب البلد ، تراوح العدد التقديري من أقل من واحد في جزر البهاما، وآيسلندا، ولكسمبرغ ومالطة إلى 350000 (فاصل عدم الثقة: 95%، 320000 إلى 380000) في الهند. وعلى المستوى الإقليمي، كانت أعلى التقديرات في منطقة جنوب شرق آسيا التابعة لمنظمة الصحة العالمية (WHO) (510000؛ فاصل عدم الثقة 95%: 450000 إلى 580000) والمنطقة الأفريقية التابعة لمنظمة الصحة العالمية (470000؛ فاصل عدم الثقة 95%: 440000 إلى 490000).

**الاستنتاج:**

لم تتم الاستفادة بالعلاج الوقائي من مرض السل في الأطفال بالشكل الكافي على مستوى العالم في عام 2017. يجب توسيع نطاق العلاج حتى يساعد في القضاء على تصاعد مرض السل وتحقيق أهداف استراتيجية القضاء على السل (End TB Strategy). ****

## 摘要

**目的:**

旨在估计 2017 年家人患有结核病且符合结核病预防治疗条件的 5 岁以下儿童人数。

**方法:**

为了估计符合条件的儿童人数，我们从已出版的资料中获取了 2017 年通过细菌学方法确诊的结核病公示案例的全国性数据、2017 年 5 岁以下人口的比例和平均家庭规模数据。通过系统综述、荟萃分析和泊松回归模型，我们获得了全球数据，关于每个家庭活动性结核病病例的数量和家人感染结核病的 5 岁以下儿童感染潜伏性结核病的患病率。

**结果:**

在 2017 年，全球符合结核病预防治疗的 5 岁以下儿童人数估计值为 127 万（95% 不确定区间，UI：1.24–1.31），这与全球儿童预防性治疗覆盖率最高为 23% 的估计值一致。从国家层面来看，估计值范围从冰岛、巴哈马群岛、卢森堡和马耳他中的任一国家的不足一人到印度的 350000 人（95% UI：320000–380000）。从区域角度来看，世卫组织东南亚地区估计值最高（510000；95% UI：450000–580000），其次是世卫组织非洲地区（470000；95% UI：440000-490000）。

**结论:**

2017 年，儿童结核病预防治疗并未在全球范围内充分实施。应扩大治疗范围，以消除感染结核病的可能性并实现“End TB”（消灭结核病）的战略目标。

## Резюме

**Цель:**

Оценка по состоянию на 2017 год количества детей младше пяти лет, проживающих в одной семье с больным туберкулезом и нуждающихся в профилактическом лечении.

**Методы:**

Для оценки количества детей, нуждающихся в лечении, авторы получили из опубликованных источников национальные показатели по состоянию на 2017 год о количестве поставленных на диспансерный учет случаев бактериологически подтвержденного туберкулеза легких, сведения о количестве детей младше 5 лет в 2017 году и средние оценки размера семьи. По результатам систематических обзоров, метаанализа и регрессионных моделей Пуассона были получены глобальные сведения о количестве случаев активной формы туберкулеза из расчета на семью с известным источником заболевания и оценки распространенности латентной туберкулезной инфекции среди детей младше 5 лет, проживающих в одной семье с больным туберкулезом.

**Результаты:**

По предварительным оценкам, во всем мире количество детей младше 5 лет, нуждающихся в профилактическом лечении от туберкулеза, составило в 2017 году 1,27 миллиона человек (95%-й интервал неопределенности, ИН: 1,24–1,31), что в лучшем случае соответствует удовлетворению потребности детей в профилактическом лечении приблизительно на 23%. Оценка распределения по странам показала разброс от менее одного ребенка в таких странах, как Багамские острова, Исландия, Люксембург и Мальта, до 350 000 (95%-й ИН: 320 000–380 000) в Индии. В региональном разрезе максимальные оценки были получены для Юго-Восточной Азии по классификации Всемирной организации здравоохранения (ВОЗ) (510 000 человек; 95%-й ИН: 450 000–580 000) и для Африканского региона ВОЗ (470 000; 95%-й ИН: 440 000–490 000).

**Вывод:**

По состоянию на 2017 год во всем мире профилактическое лечение от туберкулеза у детей применяется в недостаточной мере. Необходимо наращивать масштабы лечения, чтобы содействовать исключению резервуаров туберкулезной инфекции и достижению целей стратегии по прекращению эпидемии туберкулеза.

## Introduction

The management of latent tuberculosis infection is a critical component of the World Health Organization’s (WHO’s) End TB Strategy. Given that between a quarter and a third of the global population is estimated to be infected with *Mycobacteria tuberculosis*, ^1^^–^^3^ the Strategy’s ambitious targets and the United Nations’ Sustainable Development Goals cannot be achieved without tackling the reservoir of latent infection.^4^ The risk of progression from tuberculosis infection to active disease is particularly high in young children, who are also at the greatest risk of severe and disseminated disease.^5^ As a result, treatment of tuberculosis infection (i.e. tuberculosis preventive treatment) is strongly recommended for children younger than 5 years who are household contacts of people with bacteriologically confirmed pulmonary tuberculosis.^6^ Accordingly, coverage of tuberculosis preventive treatment is one of the key indicators used to monitor the implementation of the End TB Strategy.^7^ In 2018, world leaders committed to providing 4 million child household contacts younger than 5 years with tuberculosis preventive treatment by 2022.^8^

A recent survey of policy and practice on latent tuberculosis infection in countries with a low tuberculosis burden and in African countries found that many lacked recording and reporting systems for infection.^9^^,^^10^ In 2016, WHO started collecting data on the number of children younger than 5 years globally who were household contacts of people with pulmonary tuberculosis and who had started tuberculosis preventive treatment.^11^ Although 118 countries, including 16 of the 30 countries with a high tuberculosis burden, reported data in 2017,^11^ there was a lack of clearly defined denominators for assessing coverage of preventive treatment, which makes planning and monitoring difficult.^12^

Consequently, the aim of this study was to use tuberculosis notification data from 2017 to estimate of the number of children younger than 5 years in individual countries who were household contacts of people with pulmonary tuberculosis and who were eligible for tuberculosis preventive treatment. This information should help countries implement and monitor preventive treatment.

## Methods

Countries with a low tuberculosis burden comprised the 113 high-income or upper-middle-income countries in which the estimated annual incidence of tuberculosis disease in 2015 was fewer than 100 cases per 100 000 population, WHO’s 2015 guidelines on the management of latent tuberculosis infection are intended primarily for these countries.^13^^,^^14^ Countries with 100 or more cases per 100 000 population were regarded as having a high tuberculosis burden.

In countries with a high tuberculosis burden, the number of children eligible for tuberculosis preventive treatment was defined as the number younger than 5 years who are household contacts (hereafter referred to as child household contacts) of people with bacteriologically confirmed pulmonary tuberculosis and who do not themselves have active tuberculosis, regardless of whether they have a confirmed tuberculosis infection (in accordance with WHO guidelines on the management of tuberculosis in children).^5^ In countries with a low tuberculosis burden, the number of children eligible for tuberculosis preventive treatment was defined as the number of children younger than 5 years who are household contacts of people with bacteriologically confirmed pulmonary tuberculosis, who do not themselves have active tuberculosis and who have a confirmed tuberculosis infection, as indicated by a positive result on a standard tuberculin skin test or an interferon-gamma release assay. Consequently, the number of child household contacts eligible for tuberculosis preventive treatment, *N*, was calculated using:
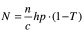
(1)in countries with a high tuberculosis burden; and
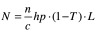
(2)in countries with a low tuberculosis burden; where *n* was the number of notified cases of bacteriologically confirmed, pulmonary tuberculosis in the country, *C* was the average number of active tuberculosis cases per household with an index case, *h* was the average household size, *p* was the proportion of the national population that was younger than 5 years, *T* was the proportion of child household contacts who had active tuberculosis, and *L* was the prevalence of a confirmed latent tuberculosis infection among child household contacts. For countries with a high tuberculosis burden, *L* was not included in the calculation because eligibility for tuberculosis preventive treatment did not depend on confirmation of infection. We did not estimate numbers for countries or territories with a population under 300 000.

Table 1 details how we derived values for the parameters in these two equations. From the literature, we obtained country-specific values of *n* and *p* for 2017, country-specific values of *h* for different years and a global estimate of *T*. To obtain a global value for *L*, we updated a recent systematic review and meta-analysis, and to obtain a global value for *C*, we carried out a new systematic review of the literature from 1 January 2005 to 11 November 2017.^18^ For both the updated and new systematic reviews, we used the reference list of Fox et al.’s systematic review,^18^ which included publications up until 1 October 2011, and supplemented it with papers subsequently published up until 11 November 2017. The new systematic review did not consider publications before 2005 because we judged that earlier publications would not reflect the current situation. The following search string was used in PubMed® for both reviews: (tuberculosis[Title] OR “tuberculosis”[MeSH Terms] OR “mycobacterium tuberculosis”[MeSH Terms] OR “tuberculosis, pulmonary”[MeSH Terms]) AND ((“contact$”[All Fields]) OR (“contact tracing”[MeSH Terms]) OR “disease outbreaks”[MeSH Terms] OR “contact*”[Title] OR “spread”[Title] OR “contact screen*”[All Fields] OR “contact tracing”[Title] OR “disease transmission”[All Fields] OR “case find*”[Title] OR (cluster*[Title] AND analys*[Title]) OR “household*”[All Fields] OR “household contact*”[All Fields] OR (“case finding”[All Fields]) OR (“casefinding”[All Fields]) OR “case detection”[All Fields]).

**Table 1:** Parameters for estimating the number of child household contacts eligible for tuberculosis preventive treatment

Parameter^a^	Value, mean (95% CI)	Source
Number of notified cases of bacteriologically confirmed pulmonary tuberculosis in 2017 (*n*)	Country-specific values (Table 4)	WHO tuberculosis burden estimates^15^
Number of active tuberculosis cases per household with an index case (*C*)	1.06 (1.04–1.07)	New systematic review of the literature from January 2005 to November 2017
Average household size (*h*)	Country-specific values^b^	National censuses, national surveys (e.g. DHSs), statistical yearbooks and official websites of national statistical authorities
Proportion of the population aged < 5 years in 2017 (*p*)	Country-specific values^b^	United Nations 2017 revision of world population prospects^16^
Proportion of child household contacts (age < 5 years) of a tuberculosis case who had active tuberculosis themselves (*T*)	6.1% (1.0–16.3)	Dodd et al., 2014^17^
Prevalence of a confirmed latent tuberculosis infection among children aged < 5 years who were household contacts of a tuberculosis case in countries with fewer than 100 cases per 100 000 population (*L*)	27.9% (18.8–39.4)	Updated systematic review of the literature from inception to November 2017

CI: confidence interval; DHS: demographic and health survey; WHO: World Health Organization.

^a^ The characters in parentheses represent the parameters in equations in the text.

^b^ Details available from the corresponding author on request.

For the updated and new systematic reviews: (i) household contacts were defined as people living in the same household or people who satisfied the definition of a household contact in the original publication; (ii) an index case was defined as the first identified case of new or recurrent tuberculosis disease in a person of any age in a specific household or as defined in the original publication; (iii) a person was defined as having a tuberculosis infection if the induration 48 to 72 hours after a tuberculin skin test was 10 mm or greater or, if this information was not available, the person satisfied the definition of a tuberculosis infection in the original publication; and (iv) a prevalent tuberculosis case was defined as a case of active disease that was diagnosed at the baseline visit during the study or within 3 months of diagnosis of the index case.

**Fig. 1:**
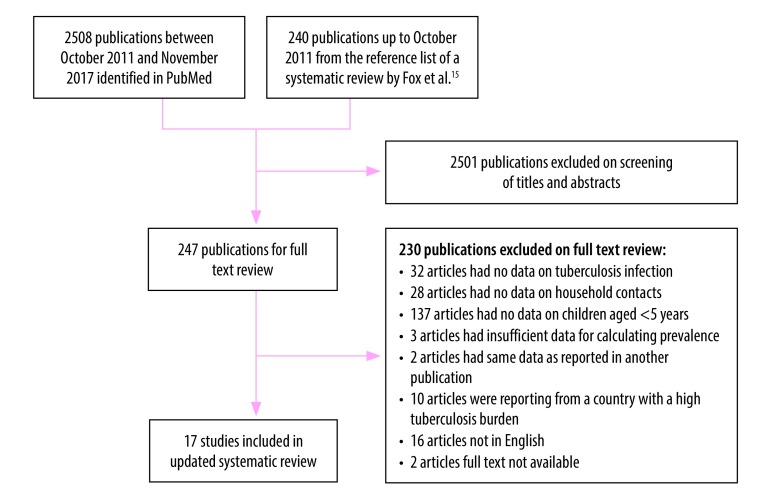
Flowchart for the selection of studies on the prevalence of latent tuberculosis infection among child household contacts, countries with a low tuberculosis burden, worldwide, 1964–2017

Notes: We defined a child household contact as a child younger than 5 years living in the same household as a person with active tuberculosis disease. A low tuberculosis burden was defined as fewer than 100 cases per 100 000 population.

To obtain a global value for *L,* we included studies in the updated systematic review that reported the prevalence of tuberculosis infection among child contacts in countries with an annual incidence of tuberculosis under 100 cases per 100 000 population at the time of the study, according to WHO estimates.^15^ If an appropriate WHO estimate was not available, we used estimates from the published literature. We also included studies that reported data on children up to 4 or 6 years of age. The reasons for excluding studies are listed in Fig. 1.

**Fig. 2:**
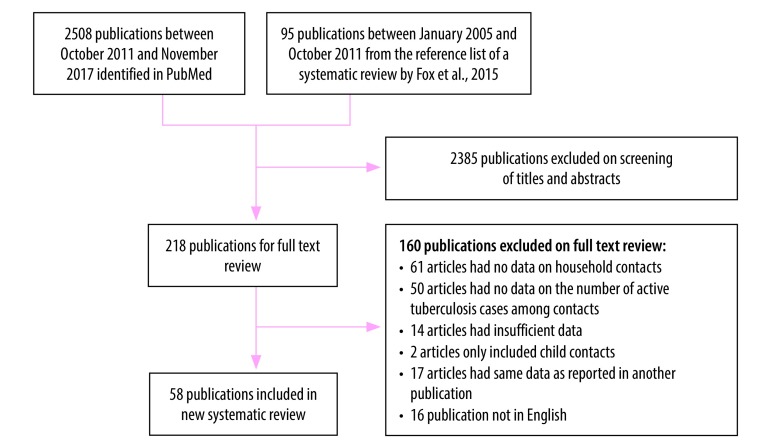
Flowchart for the selection of studies on active tuberculosis cases in households with an index case, worldwide, 2005–2017

To obtain a global value for *C*, we included studies in the new systematic review that reported the number of index tuberculosis cases, the number of household contacts and the number of prevalent active tuberculosis cases among household contacts. We excluded studies if: (i) data on contacts other than household contacts were included; (ii) the number of cases or household contacts was less than 10; (iii) only child contacts were included (this would have led to an underestimate of the number of active tuberculosis cases in the household); or (iv) the study was not published in English (Fig. 2).

One author screened all titles and abstracts for relevance and then reviewed the full text of all potentially eligible articles. For both reviews, we extracted information on the country’s name, the year of the study, the definitions of index cases and household contacts, and the number of household contacts. For the updated systematic review, we obtained information about the number of child household contacts with a confirmed latent tuberculosis infection, the tuberculin skin test cut-off criterion for infection in a child contact, the child’s bacillus Calmette–Guérin (BCG) vaccination status and the age of index cases. For the new systematic review, we extracted information on the age and number of index cases and the number of active tuberculosis cases among household contacts. In evaluating the quality of individual studies, we used a checklist modified from an existing tool to assess issues related to contact investigations and tuberculosis infection.^19^

### Data analysis

The meta-analysis of the prevalence of a confirmed latent tuberculosis infection among child household contacts (*L*) was conducted using a logistic-normal random-effects model.^20^ In the primary analysis, we did not consider the different definitions of tuberculosis infection used in the studies. The heterogeneity of study findings was assessed by visual inspection of forest plots and from the results of likelihood-ratio tests. Potential sources of heterogeneity were investigated in subgroup analyses that considered the following factors: (i) whether the index case tested positive or negative on smear microscopy; (ii) the tuberculin skin test cut-off value (i.e. 10 mm or more versus other values); (iii) the year of study publication (i.e. before 2000 or later); (iv) the country’s income status (i.e. whether high- or upper-middle-income);^21^ and (v) BCG vaccination coverage.

The average number of active tuberculosis cases per household with an index case (*C*) was estimated as follows. For each study, the average number of active tuberculosis cases among contacts in each household was calculated by dividing the number of prevalent active tuberculosis cases among household contacts by the number of index cases, which was assumed to be equal to the number of households. Data were pooled using mixed-effects, Poisson regression models. Subsequently, the average number of tuberculosis cases per household was calculated as the pooled average number of tuberculosis cases among contacts in each household plus one to account for the index case. The heterogeneity of study findings was assessed by visual inspection of forest plots and the effect of the national tuberculosis burden on estimates was assessed in a subgroup analysis. We also conducted a sensitivity analysis by excluding an outlier value for the number of tuberculosis cases per household to assess its influence on the pooled estimate.

We did not evaluate publication bias using statistical tests (e.g. Begg’s test or Egger’s test) or funnel plots because their utility has not been established in the meta-analyses of proportions obtained from observational studies.^18^^,^^22^ We considered uncertainty in: (i) the prevalence of tuberculosis infection in child contacts; (ii) the number of tuberculosis cases per household; and (iii) the proportion of child household contacts with active tuberculosis disease. We ignored uncertainty in population size estimates from the United Nations Population Division. Errors were propagated using a second-order Taylor series expansion.^23^^,^^24^ All statistical analyses were performed using Stata v. 13.1 (StataCorp LP., College Station, United States of America) and R v. 3.4.4 (The R Foundation, Vienna, Austria).

## Results

**Fig. 3:**
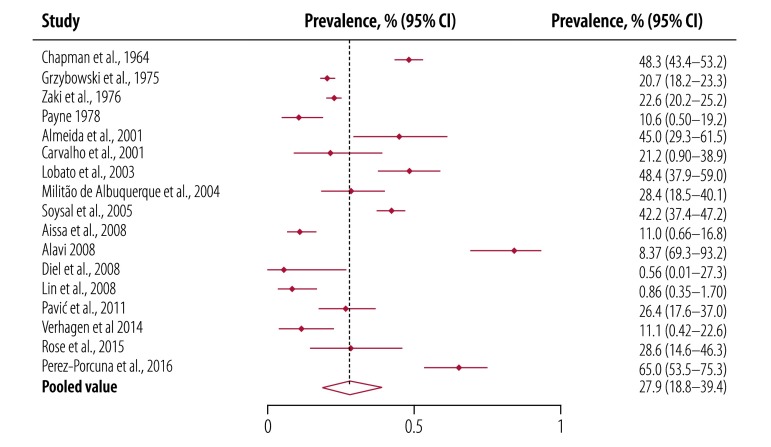
Forest plot of the prevalence of latent tuberculosis infection among child household contacts, countries with a low tuberculosis burden, worldwide, 1964–2017

Our systematic review of the prevalence of a latent tuberculosis infection among child household contacts younger than 5 years (*L*) in countries with a low tuberculosis burden included 17 studies (Fig. 1 and Table 2).^25^^–^^41^ Nine of the 17 (52.9%) were conducted in high-income countries. The presence of a tuberculosis infection was defined as an induration of 10 mm or more on the tuberculin skin test in 11 studies, whereas the other six used different criteria: (i) one used an induration cut-off of 5 mm; (ii) three used multiple induration cut-offs, ranging from 5 to 15 mm depending on BCG vaccination status, the infectiousness of the index case or the study site; (iii) one used a Heaf grade of 2, 3 or 4; and (iv) one did not specify the criterion. The median prevalence of latent tuberculosis infection among child contacts was 26.4% (interquartile range: 11.1–42.2). Twelve studies included children who had received a BCG vaccination, one included only unvaccinated children and BCG vaccination status was not specified in four studies. There was substantial heterogeneity across the studies. The pooled prevalence of latent tuberculosis infection among child contacts younger than 5 years was 27.9% (95% confidence interval, CI: 18.8–39.4; Fig. 3). None of the subgroup analyses found significant differences between subgroups.

**Table 2:** Systematic review of the prevalence of latent tuberculosis infection among child household contacts,^a^ countries with a low tuberculosis burden,^b^ worldwide, 1964–2017

Study reference	Country	Year of study enrolment	Definition of index tuberculosis case	Prevalence of latent tuberculosis infection among child household contacts aged < 5 years, no. infected children/no. all children (%)	Criterion for tuberculosis infection	BCG vaccination status
Chapman et al., 1964^25^	United States	NA	Pulmonary tuberculosis (no information on bacteriological status)	200/414 (48.3)	Not defined	Unknown
Grzybowski et al., 1975^26^	Canada	1966–1971	Pulmonary or extrapulmonary tuberculosis	209/1012 (20.7)	Tuberculin skin test induration ≥ 6 mm or ≥ 10 mm, depending on study site	Unknown
Zaki et al., 1976^27^	United States	1965–1972	Pulmonary tuberculosis (no information on bacteriological status)	254/1122 (22.6)	Tuberculin skin test induration ≥ 10 mm	Unknown
Payne, 1978^28^	United Kingdom	1968–1974	Pulmonary or extrapulmonary tuberculosis	9/85 (10.6)	Heaf grade 2, 3 or 4	No children vaccinated
Almeida et al., 2001^29^	Brazil	1998	Smear-positive pulmonary tuberculosis	18/40 (45.0)	Tuberculin skin test induration ≥ 10 mm	No specific data for children aged < 5 years; 81% of the study population vaccinated
Carvalho et al., 2001^30^	Brazil	1995–1997	Smear-positive pulmonary tuberculosis	7/33 (21.2)	Tuberculin skin test induration ≥ 10 mm	No specific data for children aged < 5 years; 75% of the study population vaccinated
Lobato et al., 2003^31^	United States	1994	Pulmonary tuberculosis (smear-positive or -negative)	45/93 (48.4)	Tuberculin skin test induration ≥ 5 mm	Unknown
Militão de Albuquerque et al., 2004^32^	Brazil	1997–1999	Pulmonary tuberculosis (including clinically diagnosed disease)	21/74 (28.4)	Tuberculin skin test induration ≥ 10 mm	No specific data for children aged < 5 years; 87% of the study population vaccinated
Soysal et al., 2005^33^	Turkey	2002–2003	Smear-positive pulmonary tuberculosis	171/405 (42.2)	Tuberculin skin test induration ≥ 10 mm	No specific data for children aged < 5 years; 79% of the study population vaccinated
Aissa et al., 2008^34^	France	2004–2005	Culture-positive pulmonary tuberculosis	18/164 (11.0)	Tuberculin skin test induration ≥ 10 mm for BCG-vaccinated people; ≥ 15 mm or conversion from negative (i.e. < 5 mm) to positive (i.e. ≥ 10 mm) for non-vaccinated people	No specific data for children aged < 5 years; 98% of the study population vaccinated
Alavi, 2008^35^	Iran (Islamic Republic of)	2003–2005	Pulmonary tuberculosis (smear-positive or -negative)	36/43 (83.7)	Tuberculin skin test induration ≥ 10 mm	No specific data for children aged < 5 years; 51% of the study population vaccinated
Diel et al., 2008^36^	Germany	2005–2006	Smear-positive pulmonary tuberculosis	1/18 (5.6)	Tuberculin skin test induration ≥ 10 mm	No specific data for children aged < 5 years; 86% of the study population vaccinated
Lin et al., 2008^37^	China	2006–2007	Smear-positive pulmonary tuberculosis	7/81 (8.6)	Tuberculin skin test induration ≥ 10 mm	No specific data for children aged < 5 years; 28% of the study population vaccinated
Pavić et al., 2011^38^	Croatia	2008–2009	Not defined	23/87 (26.4)	Tuberculin skin test induration ≥ 10 mm	All children vaccinated
Verhagen et al., 2014^39^	Venezuela (Bolivarian Republic of)	2010–2011	Culture-positive pulmonary tuberculosis	6/54 (11.1)	Tuberculin skin test induration ≥ 10 mm	76% of children aged < 5 years vaccinated
Rose et al., 2015^40^	Canada	2008–2010	Culture-positive pulmonary tuberculosis	10/35 (28.6)	Tuberculin skin test induration ≥ 5 mm for contacts of a smear-positive tuberculosis case and ≥ 10 mm for contacts of a smear-negative tuberculosis case	25% of children aged < 5 years vaccinated
Perez-Porcuna et al., 2016^41^	Brazil	2009–2010	Pulmonary tuberculosis (smear-positive or -negative)	52/80 (65.0)	Tuberculin skin test induration ≥ 10 mm	All children vaccinated

BCG: bacillus Calmette-Guérin; NA: not available.

^a^ We defined a child household contact as a child younger than 5 years living in the same household as a person with active tuberculosis disease.

^b^ We defined a low tuberculosis burden as fewer than 100 cases per 100 000 population.

CI: confidence interval.

Notes: We defined a child household contact as a child younger than 5 years living in the same household as a person with active tuberculosis disease. A low tuberculosis burden was defined as fewer than 100 cases per 100 000 population.

Our systematic review of the number of active tuberculosis cases per household with an index case (*C*) included 58 studies (Fig. 2 and Table 3).^35^^,^^37^^,^^42^^–^^97^ Of the 58, 16 (27.6%) were conducted in countries with a low tuberculosis burden. The number of active tuberculosis cases among contacts in each household ranged from 0 to 0.33, except for one study that reported a value of 0.93.^35^ The pooled number of active tuberculosis cases among contacts in each household was 0.06 (95% CI: 0.04–0.07). Consequently, the average number of active tuberculosis cases per household was 1.06 once the index case had been included. There was no significant difference between countries with a low or high tuberculosis burden (*P* = 0.33). Furthermore, excluding the one outlier reduced the average number of cases per household by only 0.002.

**Table 3:** Systematic review of active tuberculosis cases in households with an index case, worldwide, 2005–2017

Study reference	Country	Year of study enrolment	Definition of index tuberculosis case	Eligible age group	No. of index cases^a^	No. of tuberculosis cases among household contacts^b^	No. of tuberculosis cases among contacts per household^b^	Total no. of tuberculosis cases per household, including the index case
Becerra et al., 2005^42^	Peru	1996–1998	Culture-positive pulmonary tuberculosis	All ages	192	10	0.05	1.05
Chee et al., 2005^43^	Singapore	2000	Culture-positive pulmonary tuberculosis	All ages	679	20	0.03	1.03
Khalilzadeh et al., 2006^44^	Iran (Islamic Republic of)	2002–2004	Smear-positive pulmonary tuberculosis	All ages	68	17	0.25	1.25
Yeo et al., 2006^45^	Canada	1996–2000	Pulmonary or extrapulmonary tuberculosis	All ages	39	4	0.10	1.10
Hussain et al., 2007^46^	Pakistan	2001–2003	Smear-positive pulmonary tuberculosis	All ages	20	0	0.00	1.00
Alavi, 2008^35^	Iran (Islamic Republic of)	2007	Pulmonary tuberculosis (smear-positive or -negative)	All ages	69	64	0.93	1.93
Hill et al., 2008^47^	Gambia	2002–2004	Smear-positive pulmonary tuberculosis	≥ 6 months	317	33	0.10	1.10
Lee et al., 2008^48^	China, Hong Kong SAR	2000	Pulmonary or extrapulmonary tuberculosis	All ages	1 635	29	0.02	1.02
Lin et al., 2008^37^	China	2006–2007	Smear-positive pulmonary tuberculosis	All ages	393	5	0.01	1.01
Borrell et al., 2009^49^	Spain	2003–2004	Pulmonary or extrapulmonary tuberculosis	All ages	717	46	0.06	1.06
del Corral et al., 2009^50^	Colombia	2005–2006	Smear-positive pulmonary tuberculosis	All ages	366	8	0.02	1.02
Kilicaslan et al., 2009^51^	Turkey	1997–2000	Smear-positive pulmonary tuberculosis	All ages	1 570	92	0.06	1.06
Machado et al., 2009^52^	Brazil	2006–2007	Pulmonary tuberculosis (including clinically diagnosed disease)	All ages	76	2	0.03	1.03
Nguyen et al., 2009^53^	Lao People's Democratic Republic	2006	Smear-positive pulmonary tuberculosis	All ages	72	4	0.06	1.06
Ottmani et al., 2009^54^	Morocco	1993–2004	Smear-positive pulmonary tuberculosis or clinically diagnosed disease	All ages	200 902	44 110	0.22	1.22
Pai et al., 2009^55^	India	2006	Smear-positive pulmonary tuberculosis	All ages	54	1	0.02	1.02
Cavalcante et al., 2010^56^	Brazil	1999–2004	Pulmonary or extrapulmonary tuberculosis	All ages	311	26	0.08	1.08
Lienhardt et al., 2010^57^	Senegal	2004–2006	Smear-positive or culture-positive pulmonary tuberculosis	All ages	206	14	0.07	1.07
Rakotosamimanana et al., 2010^58^	Madagascar	2004–2005	Smear-positive pulmonary tuberculosis	≥ 1 year	85	12	0.14	1.14
Sia et al., 2010^59^	Philippines	2001–2008	Smear-positive pulmonary tuberculosis	All ages	218	20	0.09	1.09
Becerra et al., 2011^60^	Peru	1996–2003	Multidrug- or extensively drug-resistant tuberculosis	All ages	693	117	0.17	1.17
Grandjean et al., 2011^61^	Peru	2005–2008	Multidrug-resistant tuberculosis	All ages	358	0	0.00	1.00
Hussain et al., 2011^62^	Pakistan	unknown	Smear-positive pulmonary tuberculosis	All ages	18	0	0.00	1.00
Singla et al., 2011^63^	India	2005–2008	Multidrug-resistant tuberculosis	All ages	58	16	0.28	1.28
Vella et al., 2011^64^	South Africa	2005–2008	Multidrug- or extensively drug-resistant tuberculosis	≥ 13 years	508	64	0.13	1.13
Whalen et al., 2011^65^	Uganda	1995–2004	Smear-positive pulmonary tuberculosis	All ages	497	49	0.10	1.10
Zhang et al., 2011^66^	China	2007	Smear-positive pulmonary tuberculosis	All ages	4 695	40	0.01	1.01
Fox et al., 2012^67^	Viet Nam	2009–2011	Smear-positive pulmonary tuberculosis	All ages	167	8	0.05	1.05
Gyawali et al., 2012^68^	Nepal	2009–2010	Smear-positive pulmonary tuberculosis	≥ 5 years	184	13	0.07	1.07
Ntinginya et al., 2012^69^	United Republic of Tanzania	2010–2011	Smear-positive pulmonary tuberculosis	≥ 5 years	80	5	0.06	1.06
Shapiro et al., 2012^70^	South Africa	2009–2009	Tuberculosis based on clinical evaluation (with or without sputum smear test or sputum culture)	All ages	749	169	0.23	1.23
Thind et al., 2012^71^	South Africa	2009–2010	Smear-positive pulmonary tuberculosis	All ages	732	127	0.17	1.17
Chamie et al., 2013^72^	Uganda	Unknown	Pulmonary tuberculosis (with or without sputum smear test)	All ages	61	13	0.21	1.21
Jones-López et al., 2013^73^	Uganda	2009–2011	Smear-positive pulmonary tuberculosis	All ages	96	1	0.01	1.01
Leung et al., 2013^74^	China, Hong Kong SAR	1997–2006	Multidrug-resistant tuberculosis	All ages	256	12	0.05	1.05
Puryear et al., 2013^75^	Botswana	2009–2011	Paediatrician-diagnosed tuberculosis	All ages	163	12	0.07	1.07
Shah et al., 2013^76^	Pakistan	2010–2011	Smear-positive pulmonary tuberculosis	All ages	3 037	490	0.16	1.16
Singh et al., 2013^77^	India	2007–2011	Smear-positive pulmonary tuberculosis	All ages	450	52	0.12	1.12
Tao et al., 2013^78^	Uganda	2002–2006	Culture-positive pulmonary tuberculosis	All ages	277	19	0.07	1.07
Yassin et al., 2013^79^	Ethiopia	2010–2011	Smear-positive pulmonary tuberculosis	All ages	2 906	69	0.02	1.02
Jia et al., 2014^80^	China	2008–2008	Smear-positive pulmonary tuberculosis	All ages	1 575	92	0.06	1.06
Jones-López et al., 2014^81^	Brazil	2008–2012	Smear-positive pulmonary tuberculosis	All ages	124	2	0.02	1.02
Loredo et al., 2014^82^	Brazil	2001–2008	Pulmonary tuberculosis (smear-positive or -negative)	≥ 15 years	626	51	0.08	1.08
Thanh et al., 2014^83^	Viet Nam	2008–2008	Smear-positive pulmonary tuberculosis	All ages	1 091	27	0.02	1.02
Zelner et al., 2014^84^	Peru	2009–2012	Pulmonary tuberculosis (including clinically diagnosed disease)	All ages	3 466	229	0.07	1.07
Chamie et al., 2015^85^	Uganda	2012–2013	Pulmonary or extrapulmonary tuberculosis	≥ 18 years	54	1	0.02	1.02
Grandjean et al., 2015^86^	Peru	2010–2013	Multidrug-resistant tuberculosis	All ages	213	5	0.02	1.02
Jerene et al., 2015^87^	Ethiopia	2013–2014	Smear-positive pulmonary tuberculosis	All ages	6 015	389	0.06	1.06
Zellweger et al., 2015^88^	Ten European countries	2009–2013	Not defined	All ages	1 023	17	0.02	1.02
Guputa et al., 2016^89^	India	2013–2014	Smear-positive pulmonary tuberculosis	All ages	133	6	0.05	1.05
Javaid et al., 2016^90^	Pakistan	2012–2015	Multidrug-resistant tuberculosis	All ages	154	51	0.33	1.33
Nair et al., 2016^91^	India	2007–2014	Smear-positive pulmonary tuberculosis	All ages	280	29	0.10	1.10
Wysocki et al., 2016^92^	Brazil	2012–2013	Pulmonary tuberculosis	All ages	213	9	0.04	1.04
Armstrong-Hough et al., 2017^93^	Uganda	2015–2016	Pulmonary tuberculosis (microbiological confirmation was required for patients aged ≥ 5 years)	All ages	293	5	0.02	1.02
Datiko et al., 2017^94^	Ethiopia	2011–2013	Smear-positive pulmonary tuberculosis	All ages	5 345	169	0.03	1.03
Fox et al., 2017^95^	Viet Nam	2014	Smear-positive pulmonary tuberculosis	All ages	212	4	0.02	1.02
Mandalakas et al., 2017^96^	Eswatini	2013–2015	Initiation of antituberculosis treatment	All ages	3 258	196	0.06	1.06
Muyoyeta et al., 2017^97^	Zambia	2013–2014	Bacteriologically confirmed tuberculosis	All ages	977	19	0.02	1.02

SAR: Special Administrative Region.

^a^ We assumed that the number of index cases was equal to the number of households studied.

^b^ We defined household contacts as people living in the same household as the index case or people who satisfied the definition of a household contact in the original publication.

Using the values we obtained for *L* and *C* with the values of other parameters from the literature (Table 1), we estimated that the number of child household contacts younger than 5 years who were eligible for tuberculosis preventive treatment in 2017 ranged from less than one in four countries (i.e. Bahamas, Iceland, Luxembourg and Malta) to 350 000 (95% uncertainty interval, UI: 320 000–380 000) in India (Table 4; available at: http://www.who.int/bulletin/volumes/96/8/18-218651). Globally, the estimated number of child contacts eligible for preventive treatment was 1.27 million (95% UI: 1.24 to 1.31). Viewed regionally, the highest estimate was for the WHO South-East Asia Region: 510 000 (95% UI: 450 000–580 000; Table 5).

**Table 4:** Child household contacts^a^ eligible for tuberculosis preventive treatment, by country, 2017

Country	No. of notified, bacteriologically confirmed, pulmonary tuberculosis cases^15^	Estimated number of child household contacts^a^ eligible for tuberculosis preventive treatment, no. (95% UI)
Afghanistan	20 946	20 000 (19 000–22 000)
Albania	210	12 (8–17)
Algeria	6 575	1 100 (720–1 600)
Angola	27 086	25 000 (23 000–27 000)
Argentina	6 042	430 (270–590)
Armenia	369	80 (73–87)
Australia	780	33 (21–46)
Austria	379	10 (6.5–14)
Azerbaijan	3 125	340 (220–470)
Bahamas	16	1.0 (0.6–1.3)
Bahrain	80	8 (5–11)
Bangladesh	144 817	55 000 (50 000–59 000)
Belarus	2 171	81 (51–110)
Belgium	563	19 (12–26)
Belize	71	8.2 (5.2–11)
Benin	2 947	2 100 (1 900–2 300)
Bhutan	440	160 (140–170)
Bolivia (Plurinational State of)	5 412	1 800 (1 700–2 000)
Bosnia and Herzegovina	479	18 (11–24)
Botswana	2 098	780 (720–850)
Brazil	49 922	3 000 (1 900–4 100)
Brunei Darussalam	179	21 (13–29)
Bulgaria	694	19 (12–26)
Burkina Faso	3 841	3 300 (3 000–3 600)
Burundi	4 728	3 600 (3 300–3 900)
Cambodia	12 049	5 600 (5 100–6 000)
Cameroon	14 515	10 000 (9 500–11 000)
Canada	1 144	39 (24–53)
Cabo Verde	178	67 (61–73)
Central African Republic	5 146	3 500 (3 200–3 800)
Chad	5 162	4 500 (4 100–4 900)
Chile	2 028	120 (77–170)
China	235 547	11 000 (6 900–15 000)
China, Hong Kong SAR	2 486	74 (47–100)
China, Macao SAR	279	13 (8–17)
Colombia	8 627	630 (400–860)
Comoros	53	38 (35–41)
Congo	3 997	2 400 (2 200–2 600)
Costa Rica	313	20 (12–27)
Côte d'Ivoire	14 311	11 000 (10 000–12 000)
Croatia	287	9 (6–13)
Cuba	517	21 (13–28)
Cyprus	39	1.5 (1.0–2.1)
Czechia	366	12 (7–16)
Democratic People's Republic of Korea	40 233	9 500 (8 700–10 000)
Democratic Republic of the Congo	98 516	85 000 (77 000–92 000)
Denmark	159	4.3 (2.7–5.8)
Djibouti	1 072	610 (550–660)
Dominican Republic	2 076	180 (120–250)
Ecuador	4 299	400 (260–550)
Egypt	3 660	1 800 (1 600–1 900)
El Salvador	3 029	950 (860–1 000)
Equatorial Guinea	893	550 (500–600)
Eritrea	770	490 (440–530)
Estonia	141	3.9 (2.5–5.4)
Eswatini	2 171	1 200 (1 100–1 300)
Ethiopia	46 148	28 000 (25 000–30 000)
Fiji	141	16 (10–22)
Finland	146	4.1 (2.6–5.6)
France	2 494	85 (54–120)
Gabon	2 301	1 100 (1 000–1 200)
Gambia	1 429	1 800 (1 700–2 000)
Georgia	1 780	390 (360–430)
Germany	3 262	74 (46–100)
Ghana	8 359	3 700 (3 400–4 000)
Greece	313	8 (5–12)
Guatemala	2 760	1 400 (1 300–1 500)
Guinea	7 737	6 900 (6 300–7 500)
Guinea-Bissau	1 769	2 100 (1 900–2 300)
Guyana	342	110 (99–120)
Haiti	10 633	4 700 (4 300–5 100)
Honduras	2 190	880 (800–960)
Hungary	333	9 (6–12)
Iceland	8	0.35 (0.22–0.48)
India	905 513	350 000 (320 000–380 000)
Indonesia	215 586	72 000 (66 000–78 000)
Iran (Islamic Republic of)	4 785	360 (230–490)
Iraq	2 676	700 (440–960)
Ireland	165	8 (5–11)
Israel	131	11 (7–15)
Italy	2 160	55 (35–75)
Jamaica	69	4 (3–5)
Japan	11 227	290 (180–400)
Jordan	179	30 (19–41)
Kazakhstan	9 489	3 300 (3 000–3 600)
Kenya	46 875	25 000 (23 000–27 000)
Kiribati	189	130 (120–140)
Kuwait	373	42 (27–58)
Kyrgyzstan	3 171	1 500 (1 400–1 700)
Lao People's Democratic Republic	3 876	2 000 (1 900–2 200)
Latvia	443	13 (8.5–18)
Lebanon	325	28 (18–39)
Lesotho	3 670	1 800 (1 600–1 900)
Liberia	3 382	2 300 (2 100–2 500)
Libya	514	68 (43–94)
Lithuania	1 004	32 (20–44)
Luxembourg	21	0.7 (0.5–1.0)
Madagascar	21 773	13 000 (12 000–15 000)
Malawi	6 984	4 600 (4 200–4 900)
Malaysia	15 888	1 400 (900–2 000)
Maldives	98	14 (9–20)
Mali	4 420	6 100 (5 500–6 600)
Malta	25	0.9 (0.6–1.2)
Mauritania	1 376	1 100 (1 000–1 200)
Mauritius	109	5.2 (3.3–7.1)
Mexico	14 883	1 300 (840–1 800)
Mongolia	1 861	690 (630–750)
Montenegro	58	2.7 (1.7–3.7)
Morocco	13 635	5 500 (5 000–5 900)
Mozambique	31 606	21 000 (19 000–23 000)
Myanmar	48 088	16 000 (15 000–17 000)
Namibia	5 867	3 200 (2 900–3 400)
Nepal	16 966	6 900 (6 300–7 500)
Netherlands	367	11 (7–15)
New Zealand	167	8 (5–10)
Nicaragua	1 676	650 (600–710)
Niger	8 288	8 800 (8 100–9 600)
Nigeria	75 980	53 000 (48 000–57 000)
North Macedonia	152	8 (5–11)
Norway	137	4.5 (2.8–6.2)
Oman	193	33 (21–45)
Pakistan	138 818	110 000 (98 000–120 000)
Panama	1 012	96 (61–130)
Papua New Guinea	3 944	2 400 (2 200–2 700)
Paraguay	1 823	740 (670–800)
Peru	19 956	6 200 (5 600–6 700)
Philippines	119 712	55 000 (51 000–60 000)
Poland	3 944	130 (81–180)
Portugal	1 112	30 (19–41)
Puerto Rico	30	1.1 (0.7–1.5)
Qatar	335	23 (14–31)
Republic of Korea	19 972	600 (380–820)
Republic of Moldova	1 880	220 (200–240)
Romania	8 686	280 (180–380)
Russian Federation	40 254	1 800 (1 100–2 400)
Rwanda	4 175	2 300 (2 100–2 500)
Samoa	13	10 (9–10)
Sao Tome and Principe	46	25 (23–27)
Saudi Arabia	1 802	230 (150–320)
Senegal	10 117	13 000 (12 000–14 000)
Serbia	781	31 (19–42)
Sierra Leone	9 674	7 700 (7 100–8 400)
Singapore	1 238	51 (32–69)
Slovakia	134	4.6 (2.9–6.3)
Slovenia	89	2.9 (1.8–3.9)
Solomon Islands	126	84 (76–91)
Somalia	7 691	7 400 (6 700–8 000)
South Africa	127 187	41 000 (37 000–45 000)
South Sudan	4 333	3 600 (3 300–3 900)
Spain	2 735	77 (48–100)
Sri Lanka	4 243	1 100 (1 000–1 200)
Sudan	7 419	6 000 (5 500–6 500)
Suriname	90	8 (5–11)
Sweden	273	9 (6–13)
Switzerland	348	10 (7–14)
Syrian Arab Republic	1 080	560 (510–610)
Tajikistan	2 820	2 100 (1 900–2 300)
Thailand	36 470	5 500 (5 100–6 000)
Timor-Leste	1 954	1 600 (1 500–1 800)
Togo	2 142	1 300 (1 200–1 400)
Trinidad and Tobago	120	6.9 (4.4–9.4)
Tunisia	956	91 (57–120)
Turkey	6 162	470 (300–650)
Turkmenistan	693	110 (69–150)
Uganda	27 039	21 000 (19 000–23 000)
Ukraine	16 561	1 900 (1 800–2 100)
United Arab Emirates	47	2.8 (1.8–3.8)
United Kingdom	2 245	82 (52–110)
United Republic of Tanzania	28 542	21 000 (19 000–23 000)
United States	5 848	230 (150–320)
Uruguay	613	30 (19–42)
Uzbekistan	5 705	2 600 (2 400–2 900)
Vanuatu	47	26 (24–28)
Venezuela (Bolivarian Republic of)	7 189	670 (420–910)
Viet Nam	57 246	16 000 (14 000–17 000)
Yemen	3 487	3 000 (2 800–3 300)
Zambia	16 115	11 000 (9 700–12 000)
Zimbabwe	13 263	7 600 (7 000–8 300)

SAR: Special Administrative Region; UI: uncertainty interval.

^a^ We defined a child household contact as a child younger than 5 years living in the same household as a person with active tuberculosis disease.

**Table 5:** Child household contacts^a^ eligible for tuberculosis preventive treatment, by region, 2017

WHO Region	No. of notified, bacteriologically confirmed, pulmonary tuberculosis cases^15^	Estimated number of child household contacts^a^ eligible for tuberculosis preventive treatment, no. (95% UI)
African	713 693	470 000 (440 000–490 000)
Of the Americas	152 730	25 000 (22 000–28 000)
South-East Asia	1 414 408	510 000 (450 000–580 000)
European	129 110	16 000 (14 000–18 000)
Eastern Mediterranean	210 073	150 000 (130 000–170 000)
Western Pacific	487 089	95 000 (83 000–110 000)
**Global**	**3 107 103**	**1 270 000 (1 240 000–1 310 000)**

UI: uncertainty interval; WHO: World Health Organization.

^a^ We defined a child household contact as a child younger than 5 years living in the same household as a person with active tuberculosis disease.

## Discussion

We estimated that 1.27 million children younger than 5 years who were household contacts of people with bacteriologically confirmed pulmonary tuberculosis were eligible for preventive treatment globally in 2017. According to the WHO *Global tuberculosis report 2018*, countries reported that 292 182 child contacts received preventive treatment in 2017, which makes the best estimate of the global coverage of preventive treatment in children only 23%.^98^

Our study has several limitations. First, our estimate of the number of child household contacts was based on the number of notified bacteriologically confirmed tuberculosis cases. However, 3.6 million of the estimated 10.0 million people with incident tuberculosis globally in 2017 were neither reported nor enrolled in tuberculosis care.^98^ Consequently, our estimates are conservative, there would be substantially more eligible child contacts if all incident tuberculosis cases were considered. Second, we used national values for the average household size and for the proportion of the population younger than 5 years to estimate the number of child contacts. It is possible that the composition of households with a tuberculosis case may have differed from the national average and thus people with tuberculosis may have lived with a different number of children younger than 5 years from the national average. Furthermore, we did not consider people with tuberculosis who lived in a prison or nursing home. Doing so would have reduced the estimated number of child contacts, especially in countries where where number of tuberculosis cases among the prison and nursing home populations was high.the prison and nursing home populations were high. Third, we used the value for the average number of tuberculosis cases per household from our new systematic review for all countries, even though it may have varied between countries.

Fourth, in our updated systematic review, we observed substantial heterogeneity across studies in the prevalence of a latent tuberculosis infection among child household contacts in countries with a low tuberculosis burden. This heterogeneity probably reflects differences between studies in characteristic, such as the study population, setting, incidence of tuberculosis, the tuberculin skin test cut-off used and BCG status. We were unable to identify the source of the heterogeneity because the number of studies included in our subgroup analyses was small. Moreover, our estimates of the number of child household contacts eligible for preventive treatment in these countries were derived using an average value for the prevalence of a confirmed tuberculosis infection among child contacts, whereas the prevalence may have varied between countries. Using country-specific values would have given more accurate estimates. Nevertheless, as countries with a low tuberculosis burden accounted for only 14% of notified tuberculosis cases globally in 2017,^14^^,^^98^ their impact on our global estimate was small.

Fifth, we assumed that children were judged eligible for tuberculosis preventive treatment according to WHO guidelines.^5^ However, eligibility criteria may have varied between countries according to national policy. Sixth, we used a value for the proportion of child household contacts of a tuberculosis case who had active tuberculosis themselves (*T*) that was derived from a modelling study in 22 countries with a high tuberculosis burden,^17^ which together accounted for 80% of the global burden. However, the prevalence of active disease among household contacts in these countries was likely to have been higher than in others. Consequently, by using this proportion, we may have underestimated the number of child household contacts without active tuberculosis disease who were, therefore, eligible for preventive treatment. Our estimates of the number of children eligible for preventive treatment need to be validated using national data on the number of child contacts from well-functioning surveillance systems or surveys. These data could also be used to assess the coverage of preventive treatment directly, which should give more accurate figures than our modelling estimates with their inherent limitations. Nevertheless, in the absence of such data, our estimates should help galvanize efforts to implement, and monitor the progress of, tuberculosis preventive treatment among child contacts.

In conclusion, using our estimate of the number of children younger than 5 years eligible for tuberculosis preventive treatment, we calculated that the coverage of preventive treatment in children in 2017 was only 23%. Despite its proven efficacy, tuberculosis preventive treatment is still being underutilized. As the End TB Strategy targets can only be achieved by addressing the pool of tuberculosis infection, urgent action is needed to scale up the implementation of preventive treatment.
